# Development and Application of Nanostructured Mn_3_O_4_ Based Sensor in the Determination of Heavy Metals in Water and Wastewater

**DOI:** 10.3390/mi17030308

**Published:** 2026-02-28

**Authors:** Vasiliki Keramari, Catherine Dendrinou-Samara, Zoi Kourpouanidou, Lambrini Papadopoulou, Aristidis Anthemidis, Stella Girousi

**Affiliations:** 1 Laboratory of Analytical Chemistry, School of Chemistry, Aristotle University of Thessaloniki, 54124 Thessaloniki, Greece; keramariv@chem.auth.gr (V.K.); anthemid@chem.auth.gr (A.A.); 2 Laboratory of Inorganic Chemistry, School of Chemistry, Aristotle University of Thessaloniki, 54124 Thessaloniki, Greece; zkourpo@physics.auth.gr; 3 Department of Mineralogy-Petrology-Economic Geology, School of Geology, Aristotle University of Thessaloniki, 54124 Thessaloniki, Greece; lambrini@geo.auth.gr

**Keywords:** heavy metals, wastewater, manganese nanoparticles

## Abstract

In this work, a novel nanostructured Mn_3_O_4_-based electrochemical sensor was developed for the determination of heavy metals in aqueous media. The Mn_3_O_4_ nanostructure was solvothermally synthesized in the sole presence of propylene glycol (PG). Under the specific synthetic conditions, PG provided surface coating and stabilization by decomposition products and/or residual PG molecules that have been adsorbed on Mn_3_O_4_ NPs surfaces, creating a thin organic layer. This imparts a negative surface charge (zeta potential), enhancing colloidal stability in dispersions and electrochemical performance. The physicochemical properties of the resulting NPs were characterized via X-ray diffraction (XRD), Fourier transform infrared (FT-IR), Thermogravimetric Analysis (TGA), and Dynamic light scattering (DLS) and ζ-potential measurements, as well as SEM imaging of the modified electrode surface, confirming its successful formation and favorable structural properties. The LODs of Cd^2+^, Pb^2+^, Zn^2+^, and Cu^2+^ for their simultaneous determination are 2.9 μg·L^−1^, 5.2 μg·L^−1^, 7.1 μg·L^−1^, and 2.5 μg·L^−1^, respectively, with relative standard deviations of about 5.24%, 4.43%, 7.74%, and 4.53%, respectively. As a result of this study, a simple, sensitive, and reproducible electrochemical sensor based on a carbon paste electrode (CPE) modified with novel synthesized manganese nanoparticles and employing voltammetric techniques was applied in water and wastewater.

## 1. Introduction

The presence of heavy metals such as cadmium (Cd), lead (Pb), copper (Cu), and zinc (Zn) in municipal waste is a major environmental concern. These metals are not biodegradable and persist in the environment, accumulate in living organisms, and cause serious effects such as nephrotoxicity, neurotoxicity, mutagenicity, and carcinogenicity [1], while chronic exposure can lead to irreversible biological damage. Municipal wastewater is a major contributor to contamination in aquatic ecosystems, and the composition of sewage sludge, the main by-product of wastewater treatment, largely reflects the chemical makeup of the incoming wastewater and the treatment methods applied. Various pollutants, including heavy metals, hydrocarbons, pesticides, nitrogen compounds, pharmaceutical residues, detergents, and phosphorus, tend to concentrate in the sludge, sometimes reaching 80–90% of their initial amounts. Releasing such sludge into water bodies can substantially increase organic matter, reduce oxygen levels, and lead to nutrient enrichment [2].

Heavy metals in municipal wastewater mainly originate from domestic activities (e.g., detergents, cosmetics, and cleaning agents), corrosion and degradation of materials (such as pipelines and metal structures), and industrial effluents [3]. Pollution by lead (Pb) and cadmium (Cd), which are highly toxic [4], is also associated with the use of fertilizers and pesticides, as well as mining and chemical processing activities [5]. These processes increase the concentration of heavy metals in soil, surface water, groundwater, and the atmosphere, enhancing their mobility and potential transport through food chains. Entry into living organisms occurs through absorption in soil, consumption of contaminated water or food, and inhalation of contaminated air, leading to bioaccumulation and biomagnification, where metal concentrations increase from lower to higher trophic levels. As a result, heavy metals pose serious ecological risks and serious health impacts to both wildlife and humans at the top of the food web [6].

Wastewater treatment processes, whether biological or chemical, often fail to completely remove pollutants, potentially causing secondary pollution after discharge [7]. Biological treatment, which uses microorganisms to degrade dissolved organic matter into biomass that can be removed by sedimentation, is widely applied because of its environmental friendliness compared to chemical methods. However, it is generally ineffective in removing toxic or non-biodegradable compounds. Common technologies include oxidation ponds, aeration lagoons, anaerobic and aerobic bioreactors, activated sludge systems, percolation or trickling filters, biological filters, rotating biological contactors, and biological nutrient removal systems [8,9]. Microbial contamination can also be caused by human or animal feces, introducing protozoa, viruses, and bacteria capable of causing disease [10].

Ensuring the safety of both people and the environment means putting strong regulatory systems in place and using reliable, affordable analytical techniques to detect even very small amounts of heavy metals in municipal and industrial waste. Being able to measure these contaminants accurately is important for understanding potential environmental risks, improving how waste is managed, and meeting international safety requirements. For this reason, organizations such as the World Health Organization (WHO), the Food and Drug Administration (FDA), and the United States Environmental Protection Agency (US EPA) have set guideline limits for how much exposure to toxic heavy metals is considered safe for humans, emphasizing the importance of monitoring these substances in waste materials [11,12].

Heavy metals can be detected using a variety of analytical technologies, including atomic absorption spectroscopy (AAS), inductively coupled plasma mass spectrometry (ICP-MS), inductively coupled plasma optical emission spectrometry (ICP-OES) [13], electrothermal AAS, high-performance liquid chromatography coupled with ICP-MS (HPLC–ICP-MS) [14], and hydride generation AAS, among others [5,15,16,17]. These methods offer high sensitivity, selectivity, and precision, enabling detection of metal ions at trace or ultra-trace levels across complex matrices such as wastewater and sludge. However, conventional instrumental techniques have inherent limitations: they typically require expensive and sophisticated equipment, highly trained personnel, and time-consuming sample pretreatment steps (including digestion, extraction, and calibration), which restrict their applicability for on-site or real-time monitoring [18]. Furthermore, the operational complexity and high maintenance costs of these systems hinder their widespread use in routine wastewater monitoring, particularly in low-resource settings.

In recent years, the demand for rapid, simple, cost-effective, and adaptable detection methods has grown. Biosensors represent a promising solution, offering advantages in practicality, portability, and operational stability compared to conventional analytical techniques [19]. By combining a biological recognition element with a suitable transducer, biosensors enable selective and sensitive detection of heavy metals even at trace levels, making them highly suitable for environmental monitoring.

Electrochemical techniques, such as anodic stripping voltammetry (ASV) and differential pulse voltammetry (DPV), have also attracted significant attention due to their low cost, speed, and capability for on-site analysis [20]. These techniques offer high sensitivity and the ability to simultaneously detect multiple metal ions. The performance of electrochemical sensors largely depends on the characteristics of the working electrode, including surface morphology, conductivity, and affinity for metal ions [21,22].

Nanomaterials have emerged as effective tools for improving electrode performance, enhancing active surface area, electrical conductivity, and adsorption capacity. Among these, manganese nanoparticles are particularly notable due to their excellent electrochemical and catalytic properties, as well as their chemical stability [23]. Integration of such nanomaterials into electrode structures can significantly improve detection sensitivity, reduce detection limits, and enhance overall stability and reproducibility of electrochemical sensors [24].

Manganese nanoparticles have emerged as highly promising nanomaterials for electrochemical sensing of heavy metal ions due to their complementary physicochemical properties. Manganese nanoparticles are characterized by a high surface-to-volume ratio, abundant active sites [25], multiple oxidation states ranging from −3 to +7, with the +2 and +4 states [26], catalytic activity [27], and tunable redox behavior, which collectively enhance electron transfer kinetics [28] and enable selective detection of target metal ions [29]. Their paramagnetic or ferromagnetic behavior further facilitates analyte preconcentration and reduces matrix interference, while the semiconducting nature of MnOx nanostructures supports efficient charge transport during electrochemical processes [30,31,32].

Mn_3_O_4_ introduces pseudocapacitive redox activity: Mn^2+^ ↔ Mn^3+^ ↔ Mn^4+^. These reversible redox transitions allow enhanced adsorption of heavy metal ions, catalytic activity, and improved electron exchange. The combination of carbon with Mn_3_O_4_ gives a double-layer capacitive adsorption through carbon, while redox-assisted adsorption and detection are provided from Mn_3_O_4_. This results in higher sensitivity for metal-ion sensing, stronger adsorption in deionization applications, and better charge storage for supercapacitors.

The polyol process through a solvothermal procedure appeared to be a versatile route for the preparation of metal-based NPs, as indicated before by us and others [33]. Polyols participate in redox, complexation, and decomposition reactions with the cations and anions of the solution and form intermediates before yielding nanoparticles [34]. The reductive activity of polyols varies with their molecular weight, while they simultaneously perform multifaceted roles as solvents, stabilizers, reducing agents, and surface-modifying agents, collectively enabling precise control over nanoparticle formation and properties [35]. Compared to more toxic polyols like ethylene glycol, propylene glycol (PG) offers superior safety, versatility with diverse metal precursors, and compatibility for fine-tuning size and shape, making it an ideal medium for scalable, morphology-controlled synthesis of metal-based NPs with applications in catalysis, energy storage, and biomedicine [31,36,37,38]. Although ultra-low detection limits are often emphasized in electrochemical sensor development, practical environmental monitoring also requires robustness, reproducibility, low cost, and ease of fabrication. In this context, manganese-based oxides offer earth-abundant, noble-metal-free alternatives to Ag- or Au-based modifiers with favorable chemical stability. Accordingly, the present study emphasizes a balanced sensing platform that combines adequate μg L^−1^ detection capability with operational stability and a facile, scalable synthesis route.

In continuation of our studies on Mn-based NPs [39], herein, a novel nanoparticle-modified electrode for the simultaneous electrochemical detection of multiple heavy metals in wastewater is presented. In particular, coated with propylene glycol, Mn_3_O_4_ nanoparticles are synthesized, and their analytical performance is being discussed in the determination of heavy metals in water and wastewater samples. This work explores how the incorporation of nanomaterials enhances the electrode’s sensitivity, selectivity, and operational stability. The influence of the nanoparticles’ physicochemical properties, including surface area, conductivity, catalytic activity, and adsorption capacity, on the electrochemical response is thoroughly examined. The findings provide a foundation for the rational design of high-performance, cost-effective, and robust electrochemical sensors for rapid and accurate monitoring of heavy metal contamination in urban wastewater and other environmental matrices [40].

## 2. Experimental

### 2.1. Materials and Methods

#### 2.1.1. Chemicals and Reagents for Synthesis of Mn_3_O_4_

All the reagents utilized for our conducted experiments were of analytical grade and were used without any further purification: manganese (III) acetylacetonate (Mn(acac)_3_) (Sigma-Aldrich, St. Louis, MO, USA, ≥99.9%), 1,2-propylene glycol (PG) (Merck KGaA, Darmstadt, Germany, ≥99%, M = 76.10 g/mol), sodium hydroxide (NaOH) (Merck KGaA, Darmstadt, Germany, M = 39.997 g/mol), ethanol denaturated (disolol) (Chem. Lab., Athens, Greece, M = 46.07 g/mol), dimethyl sulfoxide (DMSO) (Sigma-Aldrich, St. Louis, MO, USA, M = 78.13 g/mol).

#### 2.1.2. Chemicals and Reagents for the Development of Sensors

All chemicals and reagents used in this study were of analytical or pro-analysis grade and applied without any further purification. All aqueous solutions were prepared using deionized water. The supporting electrolyte employed for anodic stripping voltammetry (ASV) was a 0.1 mol·L^−1^ acetate buffer solution (pH 4.6), prepared from acetic acid and sodium acetate (CH_3_COOH/CH_3_COONa, ACS reagent, Darmstadt, Germany) supplied by Merck. Standard nitrate solutions containing cadmium (Cd), lead (Pb), copper (Cu), and zinc (Zn) were purchased from Sigma-Aldrich (Darmstadt, Germany). A PTFE magnetic stirring bar (8 × 3 mm, Heinz Herenz Hamburg, Hamburg, Germany) was employed to ensure proper solution agitation during measurements.

##### Solutions

All solutions were prepared using doubly distilled water and handled at ambient temperature. The pH of each solution was carefully adjusted when necessary and monitored using a Consort C830 pH meter (Turnhout, Belgium). Prior to measurements, the electrochemical cells (25 mm diameter) were thoroughly cleaned with diluted nitric acid and rinsed several times with deionized water to avoid contamination. Nanoparticles and buffer solutions were freshly prepared and sonicated using an ultrasonic bath (TRANSONIC 460/H, Elma Schmidbauer GmbH, Singen, Germany) to ensure complete dispersion, dissolution, and homogeneity before use.

##### Apparatus

The electrochemical measurements were performed using a PalmSens Model 1 potentiostat/galvanostat (Echo Chemie, Utrecht, The Netherlands). The experimental setup consisted of a three-electrode electrochemical cell composed of a carbon paste working electrode (CPE) housed in a PTFE sleeve (3 mm inner diameter, 9 mm outer diameter), an Ag/AgCl reference electrode saturated with 3 mol·L^−1^ KCl, and a platinum wire counter electrode (Metrohm, Herisau, Switzerland). The pH values were measured using a Consort C830 pH meter (Turnhout, Belgium). Sample weighing was carried out using Sartorius-type analytical balances (Kernew 220-30014 and Denver Instrument XE-310, East Lyme, CT, USA).

##### Synthesis of Mn_3_O_4_@PG

Mn_3_O_4_@PG NPs: 0.2 g Mn(acac)_3_ was dissolved in 4 mL of PG. Separately, 0.2 g of NaOH was dissolved in 4 mL of PG. The two solutions were thoroughly mixed. The resulting solution was stirred for 10 min and then transferred into a 23 mL Teflon-lined vessel, which was sealed inside a stainless-steel autoclave to set out a solvothermal polyol process [41]. The autoclave was placed in an oven and heated to 200 °C for 24 h. The heating rate was 4 °C/min until the final temperature was reached. After 24 h, the temperature of the vessel decreased at a constant rate of 5 °C/min, remaining inside the oven until it reached room temperature. After unsealing, the material was purified through repeated washing cycles with disolol and centrifugation (20 min, 5000 rpm), where the supernatants were discarded, and a brown precipitate was acquired. Finally, the material was dried using a vacuum evaporator, and Mn_3_O_4_ nanoparticles were obtained.

#### 2.1.3. Procedures

##### X-Ray Diffraction (XRD)

The crystal structure and crystallite size of synthesized NPs were investigated through X-ray diffraction (XRD) using a Siemens Diffraktometer D5000 (Siemens AG, Karlsruhe, Germany) performed in the 2*θ* region from 10 to 70°, with monochromatized Cu-Ka X-ray radiation (λ = 1.5418 Å) and a curved crystal graphite monochromator operating at 45 kV and 100 mA; counts were accumulated every 0.02° (2*θ*) with a counting time of 2 s per step.

##### Fourier Transform Infrared Spectroscopy (FTIR)

Fourier-transform infrared (FT-IR) spectroscopy was performed using a Nicolet iS20 FT-IR spectrometer in the wavenumber range of 4000–400 cm^−1^ (Thermo Fisher Scientific, Waltham, MA, USA) equipped with a monolithic diamond, attenuated total reflection (ATR) crystal.

##### Thermogravimetric Analysis (TGA)

TGA was performed using SETA-RAM SetSys-1200 (SETARAM Instrumentation, Caluire, France) under N_2_ atmosphere, from room temperature to 900 °C with a heating rate of 10 °C/min. The percentage weight loss was used to evaluate the presence of organic coating.

##### Dynamic Light Scattering (DLS) and Zeta Potential

The surface charge was determined by DLS and zeta potential measurements, carried out using a Zetasizer Nano ZS (Malvern Instruments Ltd., Malvern, UK). Results are averages of three measurements per sample.

##### Scanning Electron Microscopy (SEM)

Sample analyses were conducted using a JEOL JSM-6390LV scanning electron microscope (JEOL Ltd., Tokyo, Japan) equipped with an INCA 300 energy dispersive X-ray spectroscopy system (Oxford Instruments Ltd., Abingdon, UK) at the Laboratory of Electron Microscopy, Aristotle University of Thessaloniki, Greece. Τhe operating conditions were at 20 kV accelerating voltage and 0.4 mA probe current, 80 s analysis time, and a beam diameter of 1 μm. For SEM observations, the samples were coated with carbon—average thickness of 200 Å—using a vacuum Agar Carbon coater (Agar Scientific Ltd., Stansted, UK).

Sample microphotographs of higher magnification were taken using a JEOL FESEM-JSM-7610 FPlus (JEOL Ltd., Tokyo, Japan) scanning electron microscope (SEM) at the Laboratory of Electron Microscopy, Aristotle University of Thessaloniki, Greece.

## 3. Voltammetric Measurements

### 3.1. Preparation of Mn_3_O_4_@PG Modified Carbon Paste Electrode (CPE) by Cyclic Voltammetry (CV)

The carbon paste electrode (CPE) was modified through electropolymerization via cyclic voltammetry to fabricate a Mn_3_O_4_@PG-CPE sensor. The electropolymerization process was carried out in 0.1 mol·L^−1^ acetate buffer (pH 4.6). The potential was cycled between −0.5 and +2.0 V for five consecutive scans at a scan rate of 0.025 V·s^−1^ with a step potential of 0.005 V.

### 3.2. Cyclic Voltammetry (CV)

For electrode modification, cyclic voltammetry (CV) was first applied to the CPE surface. The CV measurements were taken for the electrochemical characterization of the electrodes in a 5 mM potassium ferricyanide [Fe(CN)_6_]^3−/4−^ solution with the parameters of 50 mV/s scan rate, from −0.25V to +0.65 V of potential range.

### 3.3. Square Wave Voltammetry (SWV)

Square wave anodic stripping voltammetry (SWASV) was employed for the determination of heavy metals in a 0.1 mol·L^−1^ acetate buffer solution (pH 4.6). A conditioning step at +0.3 V for 30 s was applied prior to each measurement. The analytes were preconcentrated at a deposition potential of −1.4 V for 60 s. The stripping voltammograms were recorded over the potential range from −1.4 V to +0.3 V (vs. Ag/AgCl) using square-wave modulation with a frequency of 10 Hz, a pulse amplitude of 15 mV, and a potential step of 5 mV. The deposition potential and accumulation time were optimized to maximize the stripping peak currents while minimizing hydrogen evolution and signal distortion.

### 3.4. Sample Preparation

The developed technique was successfully applied to the determination of lead, cadmium, zinc, and copper in certified reference water samples with the following characteristics: BCR 714 (influent wastewater) and SRM 1643 e (simulates the elemental composition of freshwater). A special pretreatment of the samples was not necessary. The sample and the buffer solution were mixed in a 1:3 ratio prior to analysis. The measurements were performed after adjustment of pH with acetic acid to pH = 4.6 prior to analysis.

## 4. Results and Discussion

### 4.1. Synthetic Aspects and Characterization

The manganese NPs were synthesized in the sole presence of PG via anhydrous solvothermal conditions within an autoclave. PG’s high boiling point of approximately 188 °C facilitates reactions at elevated temperatures under increased pressure, preventing premature solvent evaporation and promoting the thermal decomposition of organic Mn-PG complexes into Mn-O-Mn nuclei that evolve into the desired mixed-valence Mn_3_O_4_ phase. Its viscosity not only enhances the solvation of Mn^2+^ and Mn^3+^ precursor ions for superior dispersion but also regulates nucleation and growth kinetics [42]. A key aspect of PG’s contribution lies in its mild reducing and self-regulating redox behavior: upon heating, PG undergoes controlled thermal oxidation to generate ketonic, aldehydic, carboxylic intermediates, which donate electrons to shift the Mn^2+^/Mn^3+^ equilibrium without over-reduction to metallic Mn^0^ or excessive oxidation to Mn_2_O_3_, thereby yielding the precise stoichiometric ratio required for stable mixed-valence hausmannite (Mn_3_O_4_, spinel structure with tetrahedral Mn^2+^ and octahedral Mn^3+^ sites). This organic, non-hydrolytic mechanism, distinct from aqueous processes [43], relies on the formation of transient Mn-PG complexes that decompose under solvothermal conditions, with the autogenous pressure to stabilize these intermediates to produce purer, more homogeneous phases compared to open-system heating.

**Figure 1:**
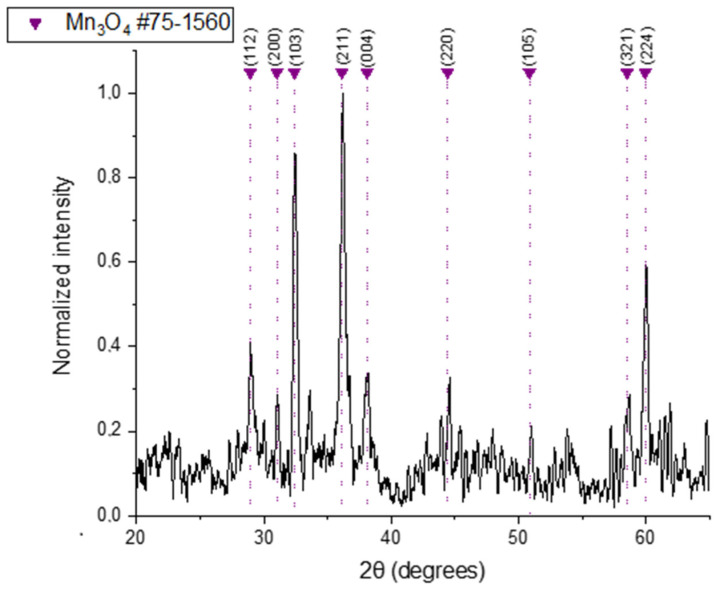
X-ray diffraction diagram of Mn_3_O_4_ @PG NPs.

XRD analysis of the Mn_3_O_4_@PG NPs (Figure 1) revealed sharp and well-defined peaks at angles (2θ) of approximately 28.9°, 31.0°, 32.3°, 36.1°, 38.1°, 44.4°, 50.8°, 59.9° and 58.5°, corresponding to the (112), (200), (103), (211), (004), (220), (105), (321) and (224) planes of Mn_3_O_4_, respectively. These diffraction peaks are consistent with standard JCPDS data (75–1560) for crystalline Mn_3_O_4_ in a tetragonal lattice structure [44]. The lattice parameters were estimated as a = b = 5.762 Å, c = 9.439 Å, and α = β = γ = 90°. The crystallite size of the NPs, calculated at 28 nm, using the Scherrer equation, D = 0.891λ/β cosθ, and based on the (211) plane.

**Figure 2:**
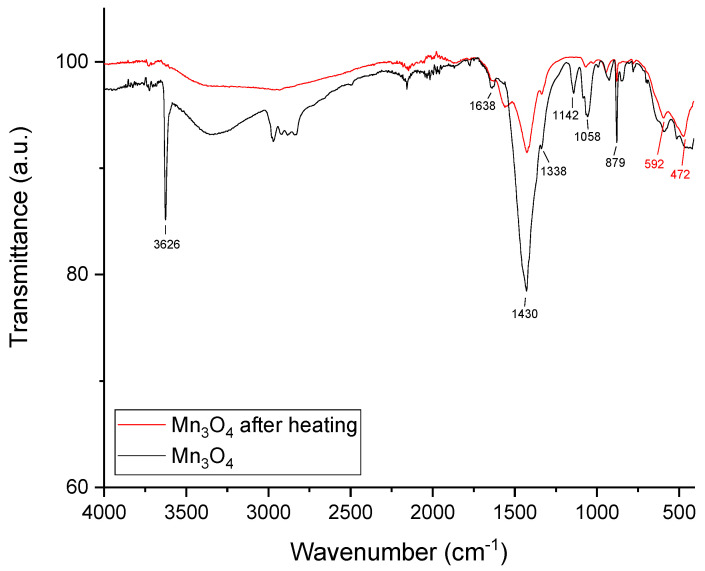
FT-IR spectra of synthesized Mn_3_O_4_@PG NPs before (black line) and after heating (red line).

The Fourier-transform infrared (FT-IR) spectrum of Mn_3_O_4_@PG NPs (Figure 2, black line) displays characteristic peaks that confirm their chemical composition. The absorption at 3626 cm^−1^ indicates O-H group stretching vibrations, suggesting the presence of hydroxyl groups of propylene glycol and/or derived products. The absorption at 1430 cm^−1^ is attributed to the stretching vibration of the C=O bond, which arises from the oxidation of propylene glycol to hydroxyacetone and lactaldehyde derivatives, while the bending vibration of the C-H bond is ascribed at 879 cm^−1^. The peak at 592 cm^−1^ is attributed to Mn-O stretching vibrations where Mn occupies tetrahedral positions, and the absorption at 472 cm^−1^ to Mn-O stretching vibrations of octahedral positions [45,46,47]. Generally, the moving force of the polyol process is the redox system. In our case, it is setting up amongst the metal precursor Mn(acac)_3_ and the PG. The mechanism follows the formation of PG-Mn complexes that decompose, giving rise to the nucleation and growth of the NPs. Both the formation and decomposition of these intermediate PG-Mn complexes are very sensitive to synthetic regulations. Thus, different oxidized derivatives are presented based on the specific synthetic conditions. However, an organic layer onto Mn_3_O_4_ NPs is evident by the FT-IR spectrum, explored further by TGA analysis.

**Figure 3:**
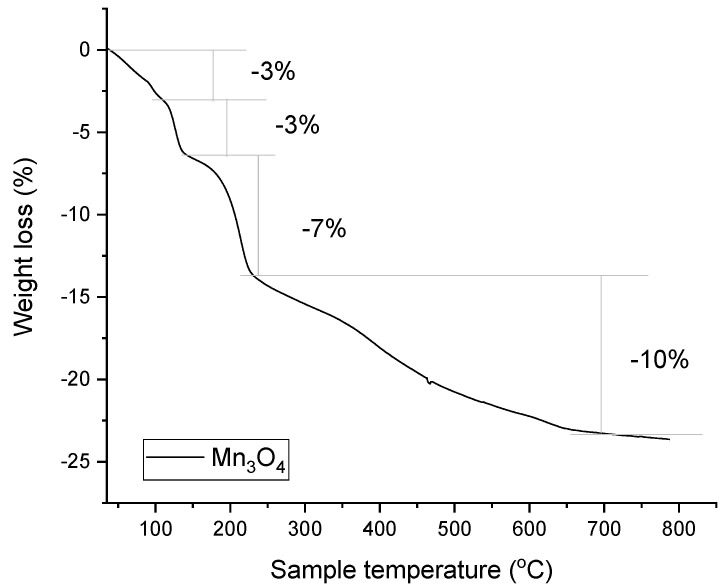
Thermogravimetric analysis of Mn_3_O_4_@PG NPs.

TGA analysis of Mn_3_O_4_@PG NPs was conducted up to 800 °C, showing four distinct stages of weight loss, as depicted in Figure 3. The first step, around 3% weight loss, occurred up to 110 °C, due to the evaporation of physically adsorbed water. From 110 to 150 °C, a double layer, about 10%, is observed, corresponding to the volatile species hydroxyacetone and lactaldehyde (PGs’ oxidation derivatives). From 150 to 800 °C, a 7% weight loss of the organic coating is observed, which is attributed to traces of chemisorbed residues from the oxidation of propylene glycol. The total weight loss of the nanoparticles reaches 23% of their initial mass. Moreover, to explore the residual decomposition byproducts of PG, we proceed to the thermal treatment of Mn_3_O_4_@PG NPs at 180 °C for 2 h in a controlled furnace atmosphere where they efficiently expel the volatile organics, hydroxyacetone, and lactaldehyde. The FT-IR spectrum after heating was recorded (Figure 2, red line), where the absence of the characteristic peak at 3626 cm^−1^ and the reduced absorption at 1430 cm^−1^ is evident. In so, a multifaceted organic surface coating onto the Mn_3_O_4_ crystallites is attributed to physisorption via weak van der Waals forces and/or hydrogen bonding (predominant for volatile species), enabling facile desorption upon heating, whereas more recalcitrant chemisorbed fragments, arising from surface-catalyzed polymerization or covalent anchoring, form a conformal organic shell. However, chemisorbed residues demand elevated temperatures (>200 °C) under inert or vacuum conditions.

**Figure 4:**
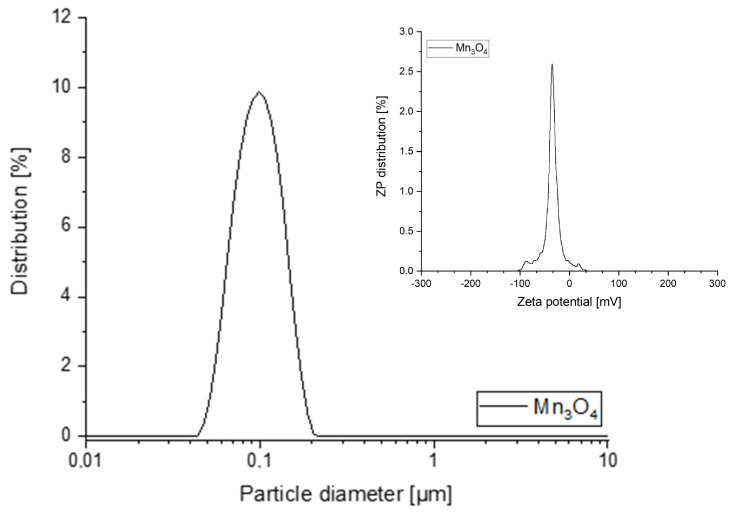
Size distribution of Mn_3_O_4_@PG NPs. Inset, ζ-potential of Mn_3_O_4_@PG NPs.

The colloidal stability of the synthesized Mn_3_O_4_@PG NPs in DMSO was confirmed by measuring their average hydrodynamic particle size and ζ-potential (Figure 4). The analysis revealed that the NPs had a hydrodynamic size of 101 nm with a polydispersity index (PDI) of 0.13 and a negative ζ-potential of −35 mV, indicating strong electrostatic repulsion and high stability against aggregation.

### 4.2. Electrochemical Characterization of the Mn_3_O_4_@PG Based Sensor

**Figure 5:**
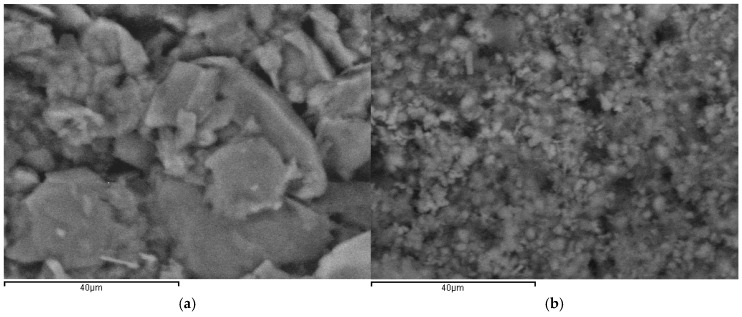
Backscattered SEM images of the electrode surface: (**a**) bare electrode exhibiting planar graphite layered morphology, and (**b**) Mn_3_O_4_@PG-modified electrode showing a granular surface structure.

The surface morphology of the electrode was investigated using scanning electron microscopy (SEM). As shown in Figure 5a, the bare electrode exhibits a surface composed mainly of planar graphite layers with a relatively compact structure. After modification with Mn_3_O_4_@PG, a markedly different surface morphology is observed (Figure 5b), displaying a granular texture with much smaller particles that cover the electrode surface. Backscattered images of higher magnification are shown in Figure 5. The surface of the bare electrode reveals stacked graphite flakes of varying sizes (Figure 5a), while clusters of manganese particles are observed on the modified electrode surface (Figure 5b).

**Figure 6:**
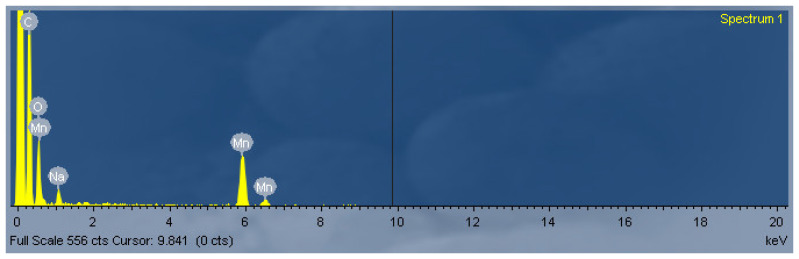
Representative energy-dispersive X-ray (EDX) spectrum.

**Figure 7:**
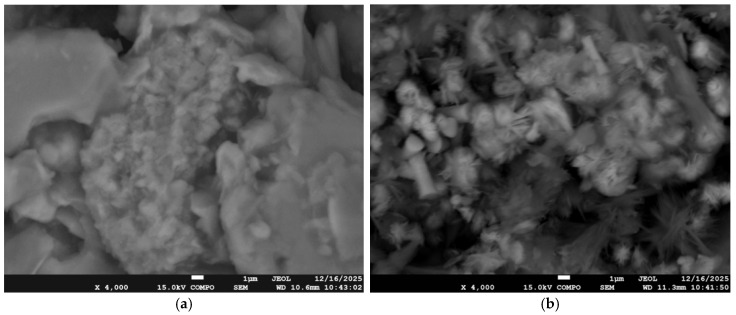
Backscattered FESEM micrographs of (**a**) bare electrode and (**b**) modified electrode surface.

In addition, the elemental composition of the synthesized Mn-based material was examined by energy-dispersive X-ray (EDX) analysis. The EDX spectrum (Figure 6) confirms the presence of manganese in the material, verifying the successful incorporation of the metal into the composite structure. The manganese content was determined from various regions of the sample, assuming a uniform elemental distribution across the surface. These findings are consistent with the SEM (Figure 7) observations and further confirm the effective morphological and compositional modification of the electrode.

**Figure 8:**
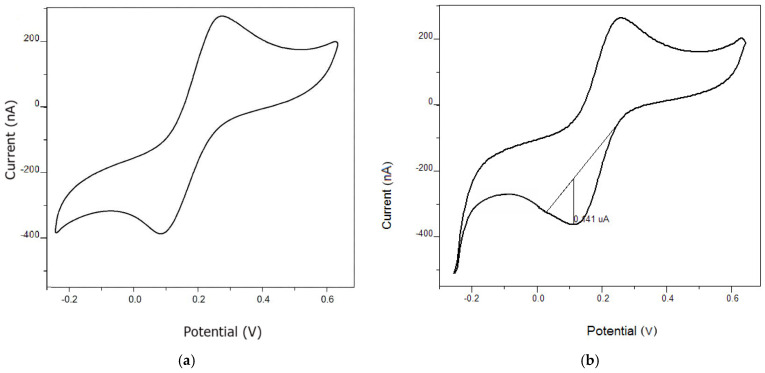
Cyclic voltammetric characterization of (**a**) the bare carbon paste electrode (CPE) and (**b**) the manganese nanoparticle–modified CPE, recorded in the potential range from −0.25 to 0.65 V at a scan rate of 0.01 V s^−1^ with a potential step of 0.005 V.

The electrochemical characterization of the bare carbon paste electrode (CPE) and the manganese nanoparticle-modified CPE surface was carried out using cyclic voltammetry (CV). The measurements were performed using a typical SCP or PSA method, as indicated in Figure 8. The resulting voltammograms illustrate the current response as a function of the applied potential, providing insight into the electrochemical behavior of the electrode surface.

### 4.3. Comparison of Bare CPE with Mn_3_O_4_ Modified CPE

**Figure 9:**
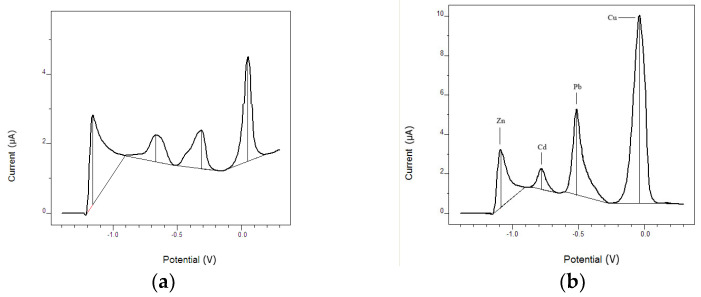
Square wave anodic stripping voltammograms comparing the response of the bare CPE (**a**) and the Mn_3_O_4_@PG-modified CPE (**b**) for the simultaneous determination of Cd(II), Pb(II), Zn(II), and Cu(II). The peaks correspond to Zn at approximately −1.0 V, Cd at −0.8 V, Pb at −0.5 V, and Cu near 0.0 V. Experimental conditions: preconcentration at −1.4 V for 60 s with 10 μg L^−1^ metal concentration; acetate buffer (pH 4.6); scan from −1.4 V to +0.2 V; frequency 10 Hz; E_step = 0.005 V; E_pulse = 0.015 V.

A clear enhancement in the electrochemical response was observed after the modification of the carbon paste electrode (CPE) with manganese nanoparticles (Mn_3_O_4_@PG). Figure 9 compares the stripping voltammograms obtained for the simultaneous detection of Cd(II), Pb(II), Zn(II), and Cu(II) using the bare and Mn_3_O_4_@PG-modified electrodes. The bare CPE exhibited relatively broad peaks with lower current intensities, whereas the Mn_3_O_4_@PG-CPE showed sharper, well-defined peaks with significantly increased peak currents for all target metals.

As clearly illustrated in Figure 9a, the bare CPE exhibits broad and partially overlapping stripping peaks in the mixed-metal solution, which limits quantitative discrimination among individual analytes. This behavior is attributed to the lack of specific adsorption sites and increased competition during deposition at the unmodified carbon surface. In contrast, modification with Mn_3_O_4_@PG (Figure 9b) significantly enhances peak currents and improves peak definition, indicating a higher electroactive surface area and more favorable metal–surface interactions.

Quantitatively, the peak current of Zn(II) increased from approximately 0.75 µA (bare CPE) to about 2–3 µA (Mn_3_O_4_@PG-CPE), corresponding to a 3–4-fold enhancement. Cd(II) increased from 1.08 µA to approximately 3–4 µA (around 3-fold), Pb(II) from 2.24 µA to 5–6 µA (about 2.5-fold), and Cu(II) from 8.15 µA to >10 µA (around 1.5-fold). This substantial increase in current response can be attributed to the larger electroactive surface area, improved catalytic activity, and enhanced preconcentration capacity provided by the Mn_3_O_4_@PG layer.

The specific surface area was estimated using the Randles–Ševčík equation, which is applicable to reversible, diffusion-controlled redox processes under semi-infinite linear diffusion conditions. The validity of these assumptions is supported by the linear dependence of the peak current on the square root of the scan rate (ν^1/2^) and the small peak-to-peak separation, indicating near-reversible electrochemical behavior and diffusion-controlled kinetics. On this basis, the use of the Randles–Ševčík equation for specific surface areaestimation is justified. The calculated electroactive surface areas were 0.0158 cm^2^ for the bare electrode and 0.0526 cm^2^ for the Mn_3_O_4_@PG-modified electrode, corresponding to an approximately 3.3-fold increase upon modification.

Overall, these findings clearly demonstrate that Mn_3_O_4_@PG modification substantially enhances both the sensitivity and selectivity of the electrochemical detection system, establishing Mn_3_O_4_@PG-CPE as a superior sensing platform for trace metal analysis relative to the unmodified electrode.

### 4.4. Analytical Performance of Mn_3_O_4_@PG Modified CPE for the Simultaneous Determination of Cd^2+^, Pb^2+^, Zn^2+^, and Cu^2+^

The analytical performance of the MnNP-modified carbon paste electrode (Mn_3_O_4_@PG-CPE) was further evaluated to assess its sensitivity and overall detection capability toward Cd^2+^, Pb^2+^, Zn^2+^, and Cu^2+^. The MnNPs-modified electrode exhibited a substantially enhanced electrochemical response compared to the bare CPE, demonstrating improved sensitivity and favorable analytical behavior for the detection of the target heavy metal ions.

Quantitative determination of metal ions was performed using external calibration based on the linear relationship between stripping peak current and analyte concentration. The analytical signal was defined as the stripping peak current (Ip, μA), measured at the maximum of each characteristic peak after baseline correction. In multicomponent systems, partial peak overlap and mutual interference are expected due to competitive deposition and stripping. Therefore, quantification was not based on assuming fully independent peaks within a single voltammogram. Instead, each analyte was quantified using calibration under fixed experimental conditions, and peak assignment and accuracy were verified by single-ion measurements, spiking experiments, and standard addition in certified reference samples to compensate for matrix effects and residual overlap.

Calibration curves were constructed according to the linear model: *Ip = a·C + b*, where Ip is the stripping peak current (μA), C is the metal ion concentration (μg L^−1^), a is the sensitivity (μA·L·μg^−1^), and b is the intercept. Linear regression parameters (a, b, and R^2^) were obtained by least-squares fitting. The limits of detection (LOD) and quantification (LOQ) were calculated using the standard deviation of the blank signal (σ) and the slope of the calibration curve (a), according to the equations: *LOD = 3σ/a* and *LOQ = 10σ/a*, where σ was determined from replicate measurements of the blank solution under identical experimental conditions.

**Figure 10:**
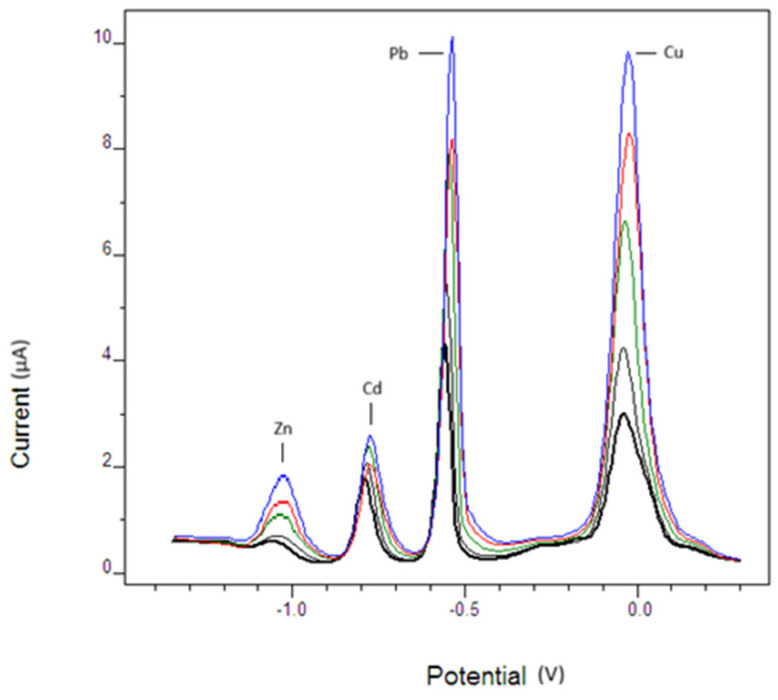
Representative square-wave anodic stripping voltammograms (SWASV) recorded using the Mn_3_O_4_@PG-modified carbon paste electrode (Mn_3_O_4_@PG-CPE) in 0.1 mol·L^−1^ acetate buffer (pH 4.6). The curves correspond to increasing concentrations of metal ions under optimized conditions (deposition potential −1.4 V, deposition time 60 s, frequency 10 Hz, step potential 5 mV, pulse amplitude 15 mV). The colored lines represent progressively increasing metal ion concentrations from the lowest (black) to the highest (blue).

Representative SWASV stripping voltammograms recorded at increasing concentrations of Cd^2+^, Pb^2+^, Zn^2+^, and Cu^2+^ under the optimized experimental conditions are shown in Figure 10. The peak currents (Ip) were obtained at the maximum of each characteristic peak after baseline correction and were subsequently used to construct the calibration plots (Ip versus concentration), from which the analytical parameters were derived.

Based on the slope of the calibration curves and the standard deviation of the blank signal, the Mn_3_O_4_@PG-CPE yielded limits of detection (LOD) of 2.9 μg L^−1^ for Cd, 5.2 μg L^−1^ for Pb, 7.0 μg L^−1^ for Zn, and 2.5 μg L^−1^ for Cu. The corresponding LOD values for the comparative electrode were 2.7, 7.1, 4.8, and 1.1 μg L^−1^, respectively. Both electrodes demonstrated low relative standard deviations (RSD < 8%), confirming satisfactory repeatability of the measurements. As shown in Table 1, the linearity coefficients (R^2^) for all analytes were high for both systems, indicating robust calibration behavior across the tested concentration range. The comparable R^2^ values suggest stable electrode–analyte interactions in both cases, while the slight differences observed in LOD can be attributed to the distinct catalytic and adsorptive properties of the Mn-based nanostructures.

Table 1 summarizes the analytical performance parameters of the Mn_3_O_4_@PG-modified carbon paste electrode for the determination of Cu^2+^, Zn^2+^, Pb^2+^, and Cd^2+^. The calibration curves exhibited good linearity, with correlation coefficients (R^2^) ranging from 0.9115 to 0.9979. The limits of detection (LOD) were found to be 2.5, 7.1, 5.2, and 2.9 μg L^−1^ for Cu^2+^, Zn^2+^, Pb^2+^, and Cd^2+^, respectively, while the corresponding limits of quantification (LOQ) were 7.6, 22.0, 16.0, and 8.8 μg L^−1^. The relative standard deviation (RSD) values, ranging from 4.43% to 7.74%, indicate good repeatability of the proposed sensor. When compared with the results reported in our previous work based on AgNPs@Sa-modified electrodes, which exhibited lower detection limits in the range of 0.38–0.72 μg L^−1^ for the same metal ions, the present Mn_3_O_4_@PG-CPE shows slightly higher LOD values [48]. Nevertheless, the proposed sensor demonstrates satisfactory analytical performance, good reproducibility, and reliable detection capability within the μg L^−1^ concentration range, highlighting its potential as an alternative nanomaterial platform for heavy metal analysis.

Accuracy and repeatability were evaluated using spiked samples at two concentration levels (4 and 28 μg/L), selected as representative low- and mid-range points of the linear working range. The modified electrode exhibited low relative standard deviations (3.65–4.47%), indicating satisfactory precision. The obtained results, together with additional analytical parameters including calibration coefficients and quantification limits, are summarized in Table 2.

The repeatability of the Mn_3_O_4_@PG-modified carbon paste electrode (Mn_3_O_4_@PG-CPE) was evaluated by three consecutive square wave anodic stripping voltammetry (SWASV) measurements (n = 3) performed under identical experimental conditions in a mixed standard solution containing Cd(II), Pb(II), Zn(II), and Cu(II). The relative standard deviation (RSD) values of the peak stripping currents were lower than 8% for all analytes, indicating acceptable short-term repeatability of the sensor.

Regenerability was examined by renewing the electrode surface after each measurement by completely replacing the carbon paste with freshly prepared Mn_3_O_4_@PG-modified paste and subsequently re-modifying the electrode before each measurement. This regeneration process was repeated for three consecutive measurement cycles, without any significant changes in the maximum currents or peak potentials. The signal retention exceeding 90% after the third cycle confirms the effective surface renewal and reliable sensor regeneration.

The modified carbon paste was prepared ex-situ immediately prior to each measurement and subsequently packed into the electrode cavity, providing a freshly renewed electroactive surface for every analysis. Consequently, the operational stability of the proposed sensor is associated with the reproducibility of the paste preparation protocol rather than prolonged surface durability. This approach ensured stable and consistent voltammetric responses throughout the study.

Overall, the Mn_3_O_4_@PG-CPE significantly enhanced the electrochemical response, yielding sharper and better-resolved stripping peaks, lower background currents, and improved signal-to-noise ratios. Additionally, the nanomodified electrode Mn_3_O_4_@PG-CPE exhibited a more balanced and reproducible performance across all four analytes, particularly with respect to peak stability and measurement consistency in standard and matrix-containing samples.

Collectively, these results indicate that the Mn_3_O_4_@PG-CPE provides strong analytical robustness, exhibiting high reproducibility, low detection limits, and stable performance in both certified freshwater samples (SRM 1643e) and complex matrix-containing samples, such as influent wastewater (BCR 714). Accordingly, it constitutes a promising sensing platform for routine environmental monitoring and trace-metal determination in waste-associated aqueous matrices.

**Figure 11:**
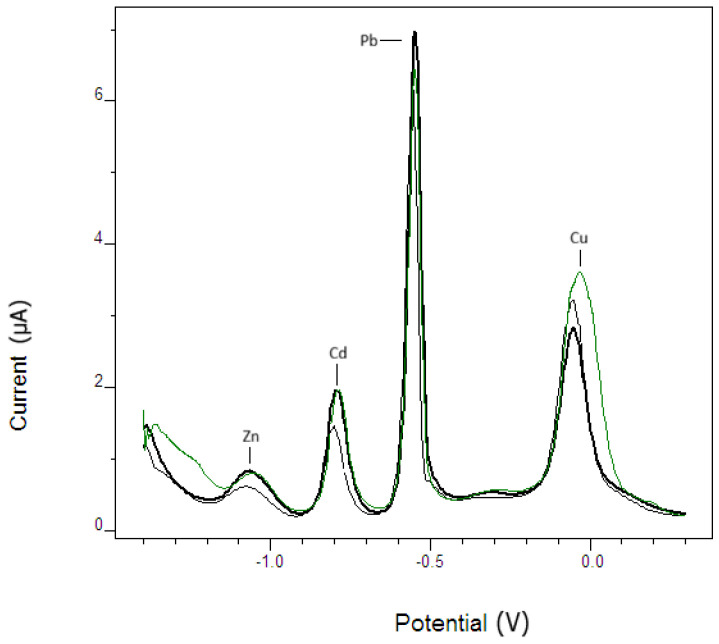
The curves correspond to the certified reference materials BCR 714 (green line), SRM 1643e (light black/gray line), and the real sample (dark black line). The strong overlap of the voltammetric profiles demonstrates good repeatability and accuracy of the method, consistent with the certified values (Table 3). Experimental conditions: E_begin = −1.4 V; E_end = 0.3 V; E_step = 0.005 V; E_pulse = 0.015 V; frequency = 10 Hz; E_cond = 0.3 V (t_cond = 30 s); E_dep = −1.4 V (t_dep = 60 s); t_equil = 0 s.

The proposed method was validated using the certified reference water samples BCR 714 (influent wastewater) and SRM 1643e (trace elements in water). Representative SWASV stripping voltammograms obtained for the two CRMs are presented in Figure 11. The peak potentials correspond to those observed in standard solutions, confirming the assignment of Cd^2+^, Pb^2+^, Zn^2+^, and Cu^2+^. Furthermore, the close overlap of the voltammetric profiles and the comparable peak currents indicate excellent method precision and accuracy, in good agreement with the certified values of the reference materials.

Table 3 summarizes the analysis of Cd^2+^, Pb^2+^, Zn^2+^, and Cu^2+^ in certified reference water samples. The close agreement between the measured and certified values, along with R.S.D. values below 5%, demonstrates the accuracy and reliability of the proposed method for real sample analysis.

### 4.5. Comparison with Recently Published Works for the Determination of Cd^2+^, Pb^2+^, Zn^2+^, and Cu^2+^

To further assess the analytical competitiveness of the proposed Mn_3_O_4_@PG-CPE, its performance was compared with that of recently reported electrochemical sensors for the simultaneous determination of Cd^2+^, Pb^2+^, Zn^2+^, and Cu^2+^.

Table 4 highlights the analytical performance of the proposed Mn_3_O_4_@PG/CPE sensor in comparison with previously reported electrochemical platforms for the simultaneous determination of Cd(II), Pb(II), Zn(II), and Cu(II). The developed method provides low limits of detection (2.9 μg/L for Cd, 5.2 μg/L for Pb, 7.1 μg/L for Zn, and 2.5 μg/L for Cu) and satisfactory repeatability (RSD < 8%), demonstrating competitive sensitivity and good precision. Although some literature-reported sensors achieve lower detection limits for specific ions, these systems often rely on more complex electrode architectures, multistep nanomaterial synthesis, or metal-film (e.g., Bi- or Hg-based) deposition procedures, which increase fabrication time, cost, and operational complexity.

In contrast, the proposed Mn_3_O_4_@PG/CPE electrode combines a straightforward and cost-effective preparation procedure with the use of environmentally friendly materials, avoiding toxic mercury films and elaborate modification protocols. Furthermore, its successful application to real wastewater samples without extensive pretreatment underlines its practical applicability in complex matrices. Overall, the proposed sensor offers a balanced combination of adequate sensitivity, good reproducibility, operational simplicity, and environmental compatibility, supporting its suitability for routine environmental monitoring and on-site analytical applications. Therefore, beyond competitive LODs, the proposed platform primarily advances practicality (facile synthesis, non-toxic composition, and matrix-robust operation), which is critical for routine wastewater monitoring.

## 5. Conclusions

In this study, the development and application of a Mn_3_O_4_@PG-based sensor for the determination of heavy metals in water and wastewater were successfully demonstrated.

The synthesized Mn_3_O_4_@PG nanocomposite exhibited favorable physicochemical and electrochemical properties, which contributed to its high sensing performance. A facile solvothermal synthetic approach has been used to prepare the coated Mn_3_O_4_ NPs through the incorporation of propylene glycol in a triple role (solvent, mild reducing agent, coating). PG inserts a moderate reducing environment for the decomposition of Mn(acac)_3_ precursor to Mn_3_O_4_ NPs, while stabilizing nanoparticles via surface coating with negative zeta potential. To our knowledge till now, bare Mn_3_O_4_ has been used before, which provides a positive surface, so the reaction conditions not only enhanced the dispersion and stability of the Mn_3_O_4_ material but also improved its surface activity, facilitating efficient interaction with target heavy metal ions.

The fabricated Mn_3_O_4_@PG-based sensor demonstrates balanced analytical performance for Cd(II), Pb(II), Zn(II), and Cu(II), combining sufficient μg L^−1^ sensitivity with good repeatability and reliable operation in both certified and matrix-rich wastewater samples. Although noble-metal modifiers can achieve lower detection limits, the present platform offers practical advantages for routine monitoring, including noble-metal-free composition, potentially lower material cost, and a facile, scalable polyol-based synthesis. The propylene glycol coating enhances colloidal stability and ensures homogeneous electrode modification, resulting in consistent electrochemical behavior. Collectively, Mn_3_O_4_@PG-CPE represents a robust and cost-effective approach for multi-metal monitoring in environmental waters under real-world conditions.

The novelty of the present work lies in the integration of solvothermally synthesized, PG-coated Mn_3_O_4_ nanoparticles into a carbon paste electrode for simultaneous multi-metal detection under real wastewater conditions. Unlike many literature-reported platforms that rely on multistep nanocomposite fabrication or in-situ Bi/Hg thin-film deposition, the proposed Mn_3_O_4_@PG-CPE is simple to fabricate, easily renewable, environmentally benign, and suitable for routine analysis. Therefore, its main advantage is not limited to analytical sensitivity, but extends to operational simplicity, reproducibility, sustainability, and practical applicability in environmental monitoring.

Overall, the Mn_3_O_4_@PG-based sensor represents a simple, cost-effective, and efficient approach for heavy metal detection in environmental samples, with significant potential for routine water quality monitoring and environmental protection applications. Future work may focus on extending the sensor’s applicability to a broader range of metal ions, improving long-term stability, and integrating the platform into portable or on-site systems for real-time analysis.

## Figures and Tables

**Table 1:** Comparison of analytical characteristics: Linearity, LOD, and LOQ, where y = Ip (μA) and x = C (μg L^−1^).

Cd	Pb	Zn	Cu	
0.0319x − 0.0686	0.0349x + 0.1153	0.0058x − 0.0192	0.0375x − 0.0527	**y**
0.9838	0.9493	0.9115	0.9979	**R^2^**
2.9	5.2	7.1	2.5	**LOD (μg/L)**
8.8	16	22	7.6	**LOQ (μg/L)**
5.24	4.43	7.74	4.53	**RSD (%)**

**Table 2:** Accuracy and repeatability obtained in the determination of zinc, cadmium, lead, and copper in standard solutions.

Metal	Added (μg/L)	Measured Concentration (μg/L)	R (%)	s_r_ % (%)
Zn	28	27.8 ± 0.6	99.3	2.13
	4	4.67 ± 0.20	116.8	4.33
Cd	28	27.9 ± 0.25	99.6	0.88
	8	8.5 ± 0.05	105.2	3.72
Pb	28	28.1 ± 0.45	100.4	1.58
	4	4.36 ± 0.07	109	3.58
Cu	28	26.9 ± 0.9	98.6	1.76
	4	4.15 ± 0.07	103.8	3.21

**Table 3:** Measurements of Cd, Pb, Zn, and Cu on two certified reference water samples.

Standard	Metal	Measured Concentration (μg/L)	R.S.D (%)	Certified Values (μg/L)
BCR 714	Cd	20.3 ± 1.7	3.1	19.9 ± 1.6
SRM 1643e		<LOQ	-	<LOQ
BCR 714	Pb	148 ± 9	3.6	145 ± 11
SRM 1643e		19.9 ± 0.3	2.8	19.63 ± 0.21
BCR 714	Zn	(1.03 ± 0.09) × 10^3^	4.2	(1.00 ± 0.10) × 10^3^
SRM 1643e		75.8 ± 1.9	3.5	76.5 ± 2.1
BCR 714	Cu	315 ± 16	4	309 ± 23
SRM 1643e		22.90 ± 0.45	2.6	22.76 ± 0.31

**Table 4:** Comparison of analytical methods for the simultaneous determination of Cd^2+^, Pb^2+^, Zn^2+^, and Cu^2+^, including the electrode type, electroanalytical technique, sample matrix, limits of detection (LOD, μg/L), relative standard deviation (RSD, %), and corresponding references.

Electrodes	Techniques	Samples	LOD (µg/L)				RSD (%)	Ref.
			Cd	Pb	Zn	Cu		
Mn_3_O_4_@PG/CPE	SWASV	Wastewater	2.9	5.2	7.1	2.5	<8	This work
G-COOH-MWCNTs/ZnO@GCE	DPV	Seawater	0.354	0.535	-	-	<9	[49]
AgNPs@Sa/CPE	SWASV	Soils & plants	0.38	0.44	0.72	0.42	<10	[48]
Bi-film/GCE	SWASV/ASV	Soils	140	30	700	380	<9	[50]
nc-Chi/GCE	SWASV	Tap water	2.15	0.891	2.82	3.64	-	[51]
GR/COF_DPTB/GCE	DPASV	Baijiu	1.24	1.81	-	0.405	<6	[52]
MnO2@RGO-NPs/GCE	DPASV	Water	0.0151	-	0.0137	0.0093	<5	[53]
BiNP/MWCNT–Na-montmorillonite/PGE	SWASV	Tap water	10.90	1.66	46.22	9.98	<8	[54]
in-situ Bi/Hg thin-film SPCE	ASV	Surface water	0.16	0.082	0.97	0.64	<4	[55]

## Data Availability

The original contributions presented in this study are included in the article. Further inquiries can be directed to the corresponding authors.
